# Recovery from trauma induced amnesia correlates with normalization of thrombin activity in the mouse hippocampus

**DOI:** 10.1371/journal.pone.0188524

**Published:** 2017-11-28

**Authors:** Marina Ben Shimon, Talya Zeimer, Efrat Shavit Stein, Avital Artan-Furman, Sagi Harnof, Joab Chapman, Arik Eisenkraft, Chaim G. Pick, Nicola Maggio

**Affiliations:** 1 Department of Neurology, The Chaim Sheba Medical Center, Ramat Gan, Israel; 2 Department of Neurology and Neurosurgery, Sackler Faculty of Medicine, Tel Aviv University, Tel Aviv, Israel; 3 Department of Neurosurgery, Rabin Medical Center, Petah Tikva, Israel; 4 Sagol School of Neuroscience, Tel Aviv University, Tel Aviv, Israel; 5 The Institute for Research in Military Medicine, The Faculty of Medicine, The Hebrew University of Jerusalem, Jerusalem, Israel; 6 Department of Anatomy, Sackler Faculty of Medicine, Tel Aviv University, Tel Aviv, Israel; 7 Talpiot Medical Leadership Program, The Chaim Sheba Medical Center, Ramat Gan, Israel; University of Toronto, CANADA

## Abstract

Transient amnesia is a common consequence of minimal traumatic brain injury (mTBI). However, while recent findings have addressed the mechanisms involved in its onset, the processes contributing to its recovery have not yet been addressed. Recently, we have found that thrombin is detected at high concentrations in the brain of mice after exposure to mTBI and that in such settings amnesia is rescued by either inhibiting thrombin activity or by blockade of PAR1. Here, we report that mice spontaneously recover from amnesia after two weeks from mTBI exposure. At this time point, long term potentiation was equally evoked in injured vs. control animals with thrombin concentration in the brain being normalized at this stage. These findings, which refer to the specific aspect of memory retrieval upon mTBI, together with our previous work, hint to a strong correlation between cognitive defects in the context of mTBI and thrombin concentrations in the brain. This may suggest that a possible scavenging of thrombin in the brain at early phases following mTBI may improve memory function.

## Introduction

Transient amnesia is a common consequence of minimal traumatic brain injury (mTBI), together with headache, dizziness, lack of concentration, tinnitus, irritability and anxiety [1]. Interestingly, while the latter symptoms may persist chronically [1, 2], amnesia seems to be reversible with most patients recovering in two weeks from the injury [3–5]. While recent findings have enriched our understanding of the mechanisms underlying amnesia following mTBI [6–9], the processes underlying its recovery have not yet been addressed.

Thrombin, a serine protease involved in the blood coagulation cascade, has been shown to affect neural function following blood-brain barrier breakdown [10–12]. Several lines of evidence exist that thrombin is also expressed in the brain under physiological conditions, suggesting an involvement of thrombin in the regulation of normal brain functions [10, 11, 13]. Among others, through the direct or indirect activation of its own receptor in the brain, the Protease-Activated Receptor-1 (PAR1), thrombin modulates synaptic transmission and plasticity [11, 14, 15] and regulates excitability [16–19] in neuronal networks.

Recently, we have found that thrombin is detected at high concentrations in the brain of mice after exposure to mTBI and that in such setting amnesia is rescued by either inhibiting thrombin activity or by blockade of PAR1 [6].

In the present study, we report that mice spontaneously recover from amnesia after two weeks upon mTBI exposure and this correlates with the normalization of thrombin concentration in the brain. These findings hint to a strong correlation between cognitive defects in the context of mTBI and thrombin concentrations in the brain. Together with our previous data, we suggest that scavenging thrombin in the brain at early phases upon mTBI may improve memory functions.

## Materials and methods

Experimental setting: The experiments were approved by the Institutional Animal Care and Use Committee of the Sheba Medical Center which obeys to national and NIH approved rules (1084/17). The minimal number of animals was used and all efforts were made to minimize suffering. The study was carried out in 8 weeks old male *ICR* mice and mTBI was induced using a free weight drop concussive device as previously described [6]. Briefly, the device consisted of an 80-cm high metal tube (13 mm in diameter) placed vertically over the head of the mouse. Minutes prior to the injury, the animals were slightly anesthetized by isoflurane (gaseous). Trauma was induced by a 30-g metal weight dropped down the metal tube on the right anterolateral side of the head (just anterior to the right ear). The mouse was placed on a sponge immobilization board which allowed head rotation following the impact thus mimicking the natural condition of head rotation in a whiplash injury. The mice were subjected to behavioral test 24 hours following the injury and 2 weeks later. Control mice underwent a similar procedure, however were un-injured. This protocol has been shown to affect hippocampal function with a minimal cellular damage [20–22].

Novel Object Recognition Paradigm: An object recognition task was used to appraise recognition memory [23]. This task takes advantage of a propensity of rodents to discriminate a familiar object from a new one. Initially, mice were individually habituated to an open field box (47×47×29 cm) for 5 min, 24 h before the test. During the acquisition phase, two objects (A and B) of identical material, which were sufficiently heavy and high to ensure that mice could neither move nor climb over them, were placed in a symmetric position within the chamber for 5-min duration. Immediately after the acquisition phase, animals underwent mTBI. 24 hours afterwards, one of the objects in the arena (A or B randomly) was substituted by a novel one (C), and exploratory behavior was again assessed for 5 minutes (discrimination phase). All objects were thoroughly cleansed (70% ethanol) between sessions to preclude odor recognition. Two weeks after mTBI, the animals were subjected to another object recognition task utilizing a new set of objects. Data acquisition and analysis was performed by EthoVision XT (Noldus, Wageningen, The Netherlands) and the behavior quantified by object nose touching at a distance of less than 4 cm and/or touching it with the nose. Successful recognition was revealed by preferential exploration of the novel object. Discrimination of visual novelty was assessed by a discrimination index defined as: (the exploration time devoted to the novel object—the time devoted to the familiar object)/(the total amount of exploration of the novel + familiar objects) [23].

Open Field Test: The open field test was performed in order to evaluate overall motor function, animals’ activity and anxiety levels. The test was performed 24 hours after the mTBI. The open field apparatus consisted of a black square field made of plastic (50 cm x 50 cm). Each mouse was placed in the corner of the apparatus at the beginning of the test and allowed to move freely as single exploration trial for 5 min. The open field was thoroughly cleaned between trials with 70% ethanol. Data acquisition and analysis was performed by EthoVision XT (Noldus, Wageningen, The Netherlands) and the behavior quantified as distance moved and the time spent in the center compared to borders region (center was defined as the 50% of the field).

Electrophysiology: Slice and electrophysiological recordings were performed as previously described [24]. Briefly, mice were rapidly decapitated, the hippocampus was removed, and 400 μm slices were prepared using a vibroslicer. Slices were then incubated for 1.5 h in a humidified, carbogenated (5% CO2 and 95% O2) gas atmosphere at 33±1°C and were perfused with ACSF [containing (in Mm) 124 NaCl, 2 KCl, 26 NaHCO3, 1.24 KH2PO4, 2.5 CaCl2, 2 MgSO4, and 10 glucose, pH 7.4] in a standard interface chamber. Recordings were made with a glass pipette containing 0.75 M NaCl (4 MΩ) placed in the stratum radiatum CA1. Stimulation was evoked using a Master 8 pulse stimulator (A.M.P.I., Jerusalem, Israel) and was delivered through a set of bipolar nichrome electrode. Long Term Potentiation (LTP) was induced by high frequency stimulation (HFS) consisting of 100 pulses at twice the test intensity, delivered at a frequency of 100 Hz (100 Hz, 1 s). Before applying the tetanic stimulation, baseline values were recorded at a frequency of 0.033 Hz. Responses were digitized at 5 kHz and stored on a computer. Offline analysis and data acquisition were performed using the Spike 2 software (Cambridge Electronic Design, UK). All numerical data are expressed as mean ± standard error of mean (SEM), and EPSP slope changes after tetanic stimulation were calculated with respect to baseline. There were no systematic differences in the magnitudes of the baseline responses in the different conditions. All values reported refer to 30 min after tetanic stimulation. Statistical evaluation was performed by either a Student's t test for paired or unpaired data as the case may be (Origin 6.0). p values of <0.05 were considered a significant difference between means.

Thrombin-Like Activity in Brain Slices: Thrombin enzymatic activity was measured using a fluorometric assay based on the cleavage rate of the synthetic substrate Boc-Asp (OBzl)-Pro-Arg-AMC (I-1560; Bachem, Bubendorf, Switzerland) and defined by the linear slope of the fluorescence intensity versus time, as previously described [25]. Two weeks following mTBI, mice were perfused with saline, the brain immediately removed and placed in steel brain matrix (1 mm Coronal, Stoelting, Wood Dale, IL, USA). The brain was then cut starting at its anterior side (starting at slice # 3, 2 mm anterior to the bregma), into coronal 1-mm thick slices. Slices 5–8 (containing the hippocampus) from the right hemisphere were collected for the assay. Slices were then placed into 96-well black microplate (Nunc; Roskilde; Denmark) containing the substrate buffer. Measurements were carried out using a microplate reader (Infinite 2000; Tecan, Männedorf, Switzerland) with excitation and emission filters of 360 ± 35 and 460 ± 35 nm, respectively. The values reported in the graph are normalized to control and represent as fold of increase (± SEM).

Quantative PCR: Prior to the harvest, the animals were anesthetized with pentobarbital and perfused transcardially with a physiological saline solution. Brains were removed and hippocampi dissected. RNA was extracted using Biorad Aurum 732–6820 (Bio-Rad Laboratories, Hercules, CA, USA) from frozen tissues. One microgram of total RNA was used for reverse transcription using high-capacity cDNA reverse transcription kit (Applied biosystems). Quantitative real-time polymerase chain reaction was performed on the StepOne™ Real-Time PCR System (Applied Biosystems, Rhenium, Israel) using Fast SYBR Green Master (ROX) (Applied biosystems). mRNA levels were examined with the following primers sequence: Factor X: 5’GTGGCCGGGAATGCAA, 5’AACCCTTCATTGTCTTCGTTAATGA, prothrombin: 5' CCGAAAGGGCAACCTAGAGC, 5' GGCCCAGAACACGTCTGTG, PAR1: 5' TGAACCCCCGCTCATTCTTTC, 5' CCAGCAGGACGCTTTCATTTTT. Hypoxanthine guanine phosphoribosyltransferase (HPRT; 5’GATTAGCGATGATGAACCAGGTT, 5’ CCTCCCATCTCCTTCATGACA) served as a reference gene in this analysis. A standard amplification program was used (1 cycle of 95°C for 20 seconds (s), 40 cycles of 95°C for 3 s and 60°C for 30 s). The results were normalized to reference gene expression within the same cDNA sample and calculated using the DCt method with results reported as fold changes relative to control brains of sham animals and graphed as mean ± SE.

Western blot detection: Hippocampus samples were homogenized in RIPA buffer [containing in mM: 50 TRIS HCl pH 8, 150 NaCl, 1% NP-40, 0.5% Sodium Deoxycholate, 0.1% SDS) and a protease inhibitor cocktail (Merck Millipore 539134). The microcentrifuge tubes were placed in a bullet blender homogenizer (Next Advance) at maximum speed for 1 minute. The homogenates were then centrifuged (13,000g X 5 min) at 4°C. The supernatants were collected and protein concentration was determined through a bicinchoninic acid (BCA) assay. 20μg from each sample were separated by SDS-polyacrylamide gel electrophoresis. The proteins were transferred onto nitrocellulose membranes. Membranes were incubated with rabbit anti FX (1:1,000, BS-77622, Bioss), Thrombin (1:400, BS-19142, Bioss), PAR-1 (1:500, BS-0828R, Bioss) over night at 4°C and washed. Membranes were then incubated at room temperature with horseradish peroxidase-conjugated goat anti-rabbit antibody (1:10,000, Jackson Immunoresearch Laboratories). Protein bands were detected by a peroxidase-based ECL method. Upon detection, the membranes were stripped and re-incubated with mouse anti-actin antibody (1:10,000, MAB1501) and re-detected by ECL. Analysis of the protein bands density was performed with ImageJ software.

## Results

**Fig 1:**
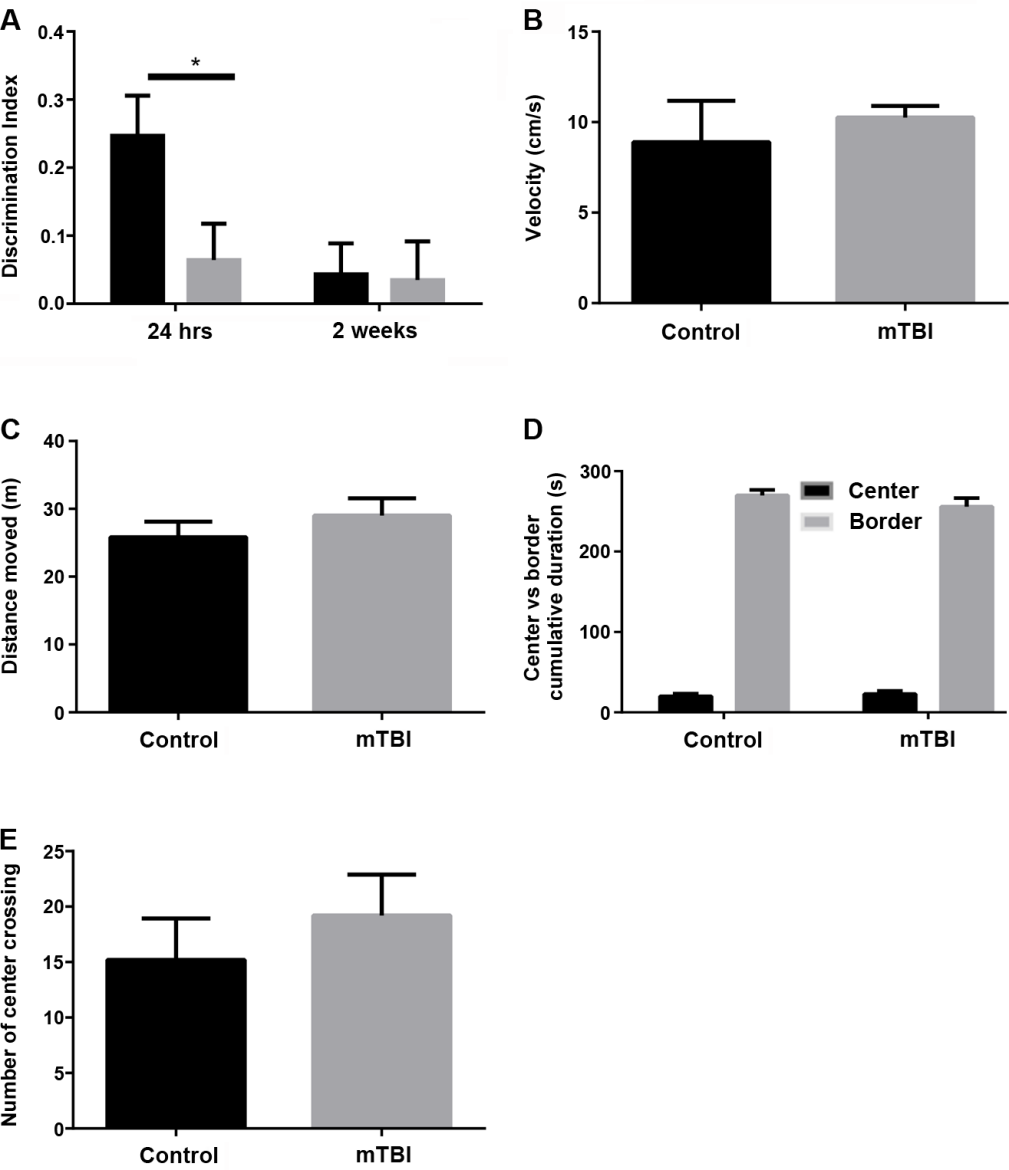
Transient amnesia following mTBI in mice. (a) The object recognition memory impairment detected 24 hours following mTBI is amended 2 weeks later. Mice were trained for 5 minutes on a novel object recognition task followed immediately by mTBI (n = 20) and compared to un-injured animals as control (n = 16). The discrimination phase was performed at 24 hours upon mTBI. Two weeks later, an additional set of object recognition tests was performed as described in the text. At this time point no difference could be detected in memory performance between the two groups of animals. Statistics reported in the text, error bars indicate standard errors. (b-e) An analysis based on the open field test aimed to assess motor activity and function/curiosity showed no differences among mTBI treated animals vs. controls.

**Fig 2:**
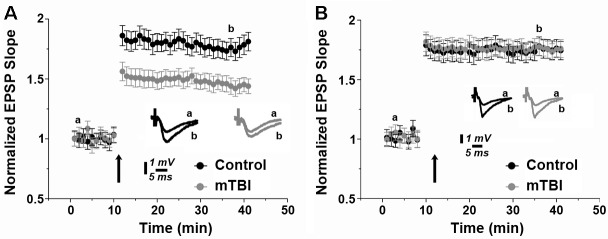
Long Term Potentiation (LTP) is equally evoked in mTBI and control animals at two weeks following injury. (a) While twenty fours upon mTBI, animals exhibited a lower LTP compared to controls., (b) no difference in LTP could be detected in mTBI exposed animals vs. control at two weeks upon injury. Sample illustrations are at the indicated time points, the arrow indicates the time of high frequency stimulation delivery.

Reversible Amnesia at two weeks after mTBI. Exposure to mTBI resulted in amnesia to a novel object recognition task tested twenty-four hours after the injury [6]. At this time point, mTBI exposed mice (n = 20) presented a discrimination index of 0.06±0.05 compared to 0.26±0.06 of controls (n = 16) (p<0.03 by two-way repeated measures ANOVA followed by Bonferroni’s multiple comparisons tests; Fig 1A). Interestingly, this trend was not statistically significant when the animals were tested two weeks following mTBI. Here, no significant difference was detected for the discrimination index between the two groups (0.03±0.04 n = 20 vs. 0.05± 0.04 n = 16 of controls, p = 0.99, Fig 1A). A two-way repeated measures ANOVA indicated no significant interaction between Time and Group variables (F (1,34) = 2.5, p>0.1) but a significant effect in each one of them (Time: F(1,34) = 4.2, p<0.05, Group: F(1,34) = 4.9, p<0.05). Additional behavioral experiments performed at the latter time point showed that neither motor curiosity neither motor function was impaired after mTBI (Fig 1B–1E). Accordingly, LTP was assessed in the two groups of animals at the different time points. At twenty four hours after the injury, mTBI exposed animals exhibited an impaired LTP (1.43 +/- 0.068 vs. 1.76 76 +/- 0.075 of controls, *p*<0.05, n = 9 slices, Fig 2A), thus confirming our previous finding [6]. However, no significant difference was detected between the two groups of animals when LTP was tested two weeks following mTBI (1.75+/- 0.077 vs. 1.76 +/- 0.071 of controls, *p =* 0.18, n = 9 slices, Fig 2B). All together these findings suggest that memory functions spontaneously recover in mice upon two weeks from mTBI.

**Fig 3:**
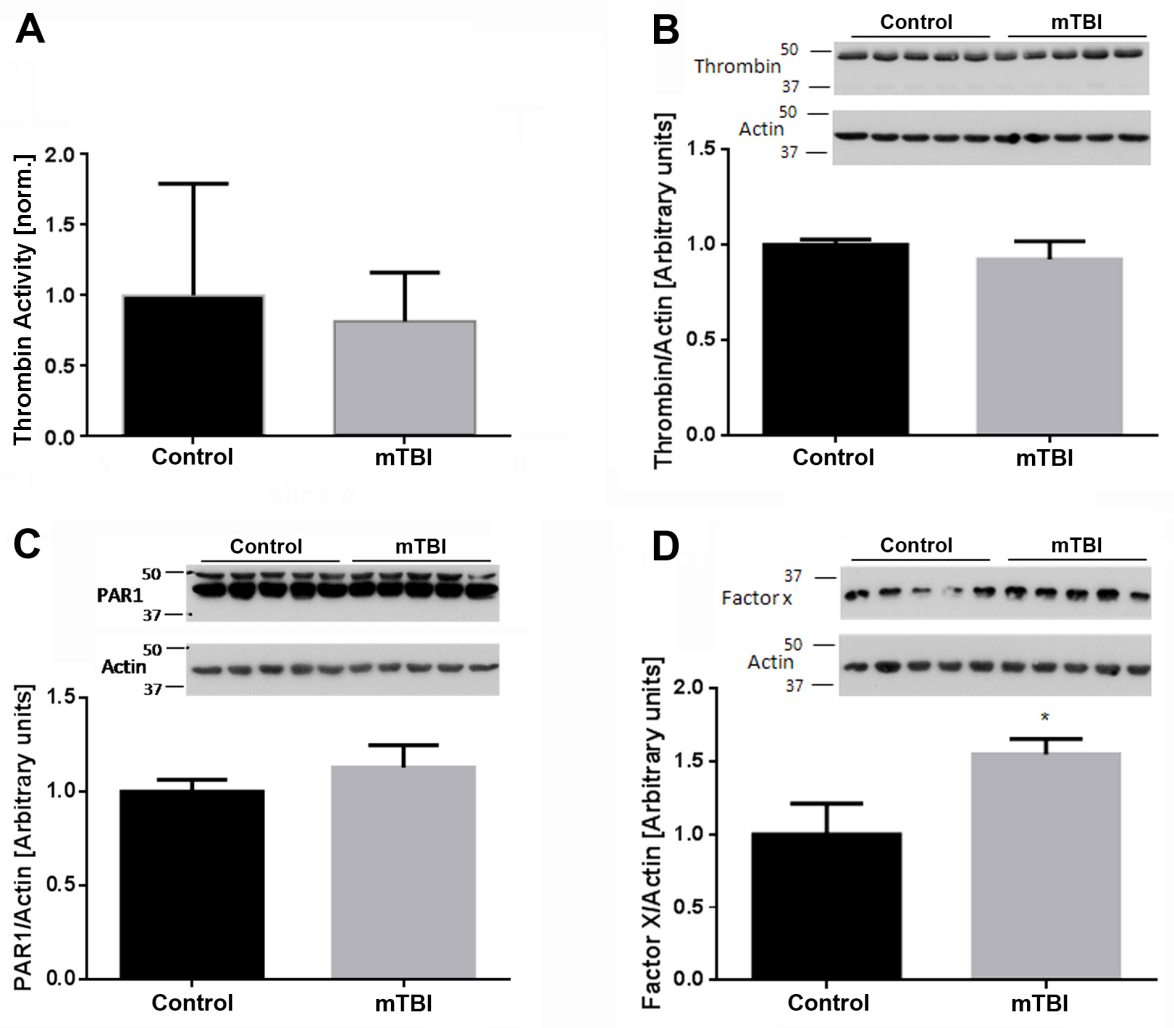
At two weeks upon injury, brain thrombin activity and concentration are similar between mTBI exposed animals and controls. (a) Thrombin activity as well as its protein levels (b) assessed as described in the methods section were comparable between mTBI and control brain slices. (c) PAR1 and (d) factor X were slightly elevated in mTBI animals compared to control. Refer to text for statistics.

**Fig 4:**
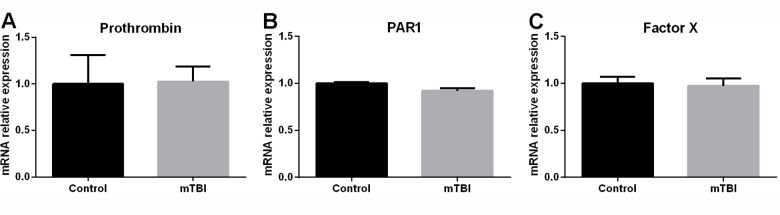
At two weeks upon injury, brain RNA levels of prothrombin, PAR1 and factor X were comparable between mTBI exposed animals and controls.

Thrombin levels normalize at two weeks after mTBI. In a previous report, we found that thrombin activity and concentration as well as PAR1 levels were significantly elevated in the brain at twenty-four hours following injury [6]. However, this was not the case when these parameters were assessed at two weeks after injury. At this time point, thrombin activity was comparable between mTBI and control brain slices (0.817±0.153 vs. 1± 0.355 of controls, p = 0.6, Fig 3A). In parallel, thrombin protein levels were similar between the two groups of animals (0.925±0.093 vs. 1± 0.027 of controls, p = 0.4, Fig 3B) while factor X and PAR1 were detected at slightly higher levels in mTBI animals (1.55±0.104 vs. 1±0.213 of controls, p = 0.049 and 1.162±0.03 vs. 1±0.04 of controls, p = 0.015, wFig 3C and 3D). Finally, RNA levels for prothrombin, PAR1 and factor X were similar between the two groups of animals (Fig 4).

## Discussion

Transient amnesia is an interesting neurological condition. When symptomatic, patients experience a striking loss of memory for recent events with a relatively impaired ability to retain new information [3]. However, people may recover completely in an undetermined time window [1, 3]. Current research has revealed that a possible hypoperfusion of the brain regions involved in learning and memory may cause neuronal dysfunction and probably cell death thus resulting in its clinical onset. [1]. However, no information currently exists on the mechanisms in charge of memory retrieval following transient amnesia. A reason for this lack of information relies on the fact that the exact time scale of memory retrieval following transient amnesia is unknown therefore reciprocal causation cannot be properly assessed. In this context, mTBI represent an interesting model to evaluate. Following mTBI, humans may experience transient amnesia with memory functions recovering in two weeks [1, 4, 5]. This specific time window gives the opportunity to address the molecular and cellular mechanisms in charge of memory loss and retrieval. The findings of the current work together with those previously published [6] show that upon mTBI, mice also experience a transient memory loss which mimics the time scale observed in the human condition. Furthermore, we now report that retrieval of memory function correlates with a normalization of thrombin concentrations in the brain. These data together with our previous findings, i.e. rescue of memory function by either blockade of thrombin or PAR1 in the acute phase of mTBI [6], suggest a possible causative role for thrombin in the pathophysiology of transient memory loss in the context of mTBI. Whether this finding may have implication for the understanding of the pathophysiology of amnesia in other neurological diseases needs still to be addressed. However, it is interesting to speculate that if thrombin has been shown to cause neuronal hyperexcitability and seizures [12, 16, 19, 26], could high thrombin concentration detected upon focal seizures with impaired awareness thus be in charge of transient amnesia following an epileptic attack? Furthermore, if both thrombin inhibitors and PAR1 antagonists may have antiepileptic properties, may these drugs be effective for accelerating memory retrieval?

Memory encompasses a wide range of functions including encoding, storing and information retrieval [27–29]. In our current work, we have assessed a specific aspect of information retrieval: the ability of relearning following mTBI. While our data suggest that relearning may recover upon two weeks from the injury, we cannot generalize these findings to other memory functions. We and others have shown that different aspects of memory are impaired at longer time scales upon mTBI both in humans and animals [4, 21, 22, 30–33]. As the physiological mechanisms underlying the different memory functions are currently unknown, so the processes in charge of its impairments need additional investigations. In addition, whether thrombin may have a role in any other aspect of memory functions is yet to be investigated.

Memory loss may either be a transient condition in some neurological settings, or it may be a persistent feature in neurodegenerative diseases. Here, a possible failure in the regulation of thrombin concentration may result in extremely high thrombin levels in the brain which may cause neuronal death [34, 35] and consequently memory loss. While this hypothesis needs to be further validated, it is interesting to speculate about the mechanisms in charge of thrombin regulation upon mTBI. In this context, thrombin concentration may rise either because of Blood Brain Barrier opening or as a result of *de novo* thrombin synthesis in the brain following injury [10, 36]. While current literature seems to support the former hypothesis [37–39], the higher levels of factor X detected upon mTBI may indicate that newly brain synthetized thrombin may also occur following mTBI.

The normalization in thrombin concentration observed two weeks after the injury may in turn follow the activity of thrombin scavengers, i.e. nexin I and II which have been previously detected in the brain [40, 41]. If this might be the case, novel treatments aiming at potentiating the activity of such proteins may be designed in order to recover memory function at an earlier time point. Nonetheless, all together our findings point to a possible correlation between cognitive defects in the context of mTBI and thrombin concentrations in the brain. Blocking thrombin activity or scavenging its concentration in the brain may improve memory at early phases upon mTBI.
